# Associations of Census-Tract Poverty with Subsite-Specific Colorectal Cancer Incidence Rates and Stage of Disease at Diagnosis in the United States

**DOI:** 10.1155/2014/823484

**Published:** 2014-08-03

**Authors:** Kevin A. Henry, Recinda L. Sherman, Kaila McDonald, Christopher J. Johnson, Ge Lin, Antoinette M. Stroup, Francis P. Boscoe

**Affiliations:** ^1^Department of Epidemiology, Rutgers School of Public Health and Rutgers Cancer Institute of New Jersey, 683 Hoes Lane West, Piscataway, NJ 08854, USA; ^2^North American Association of Central Cancer Registries, 2121 West White Oaks Drive, Suite B, Springfield, IL 62704, USA; ^3^Department of Geography, University of Utah, 260 South Central Campus Drive Room 270, Salt Lake City, UT 84112-9155, USA; ^4^Cancer Data Registry of Idaho, 615 North 7th Street, P.O. Box 1278, Boise, ID 83701, USA; ^5^Department of Health Services Research and Administration, University of Nebraska Medical Center, College of Public Health, Nebraska Medical Center, Omaha, NE 68198-4350, USA; ^6^Department of Epidemiology and Biostatistics, School of Public Health, University of Albany, State University of New York, Albany, One University Place, Rensselaer, NY 12144, USA

## Abstract

*Background*. It remains unclear whether neighborhood poverty contributes to differences in subsite-specific colorectal cancer (CRC) incidence. We examined associations between census-tract poverty and CRC incidence and stage by anatomic subsite and race/ethnicity. *Methods*. CRC cases diagnosed between 2005 and 2009 from 15 states and Los Angeles County (*N* = 278,097) were assigned to 1 of 4 groups based on census-tract poverty. Age-adjusted and stage-specific CRC incidence rates (IRs) and incidence rate ratios (IRRs) were calculated. Analyses were stratified by subsite (proximal, distal, and rectum), sex, race/ethnicity, and poverty. *Results*. Compared to the lowest poverty areas, CRC IRs were significantly higher in the most impoverished areas for men (IRR = 1.14 95% CI 1.12–1.17) and women (IRR = 1.06 95% CI 1.05–1.08). Rate differences between high and low poverty were strongest for distal colon (male IRR = 1.24 95% CI 1.20–1.28; female IRR = 1.14 95% CI 1.10–1.18) and weakest for proximal colon. These rate differences were significant for non-Hispanic whites and blacks and for Asian/Pacific Islander men. Inverse associations between poverty and IRs of all CRC and proximal colon were found for Hispanics. Late-to-early stage CRC IRRs increased monotonically with increasing poverty for all race/ethnicity groups. *Conclusion*. There are differences in subsite-specific CRC incidence by poverty, but associations were moderated by race/ethnicity.

## 1. Introduction

Colorectal cancer (CRC) is made up of a set of cancers that develop in different physical locations of the colon and rectum, including the proximal colon, distal colon, and rectum. These anatomic subsites are heterogeneous in terms of incidence, etiology, clinical and pathological characteristics [1], recommended treatments, and outcomes [2–5]. In the United States (US), cancers in the proximal colon account for the greatest burden of CRC (21.1 cases per 100,000), followed by the rectum (14.1 cases per 100,000) and the distal colon (13 cases per 100,000) [6].

Subsite-specific incidence rates also vary by race and ethnicity. US studies indicate that incidence rates of proximal and distal colon cancer are higher among blacks than whites, whereas rectal cancer rates are higher among whites compared to blacks [7–9]. Among Asians and Pacific Islanders (API), CRC rates are significantly lower than in whites and blacks across most anatomic subsites except for rectal cancer, where the incidence rate in API males is higher compared to black males [8]. The reasons for these variations are not fully understood but may be related to genetic or behavioral factors, including diet and uptake of preventive screening [10].

It has also been reported that CRC and subsite-specific incidence rates vary by socioeconomic status (SES), although the direction of the association varies globally. In the USA and Canada, lower SES has been associated with a higher risk of CRC, whereas in Europe, Australia, and South Korea, lower SES has been associated with a lower risk of CRC [11, 12]. While SES is not a direct determinant of incidence differences by subsite, variance in incidence rates among SES groups is likely due to common CRC risk factors which vary by SES, such as physical inactivity, unhealthy diet, smoking, obesity, and poor access to and underuse of screening services for early detection and removal of precancerous polyps [13].

We are aware of only two population-based US studies that have examined the role of SES on CRC incidence by anatomic subsite and race/ethnicity [12, 14]. In a study of CRC cases diagnosed in the USA from 1999 to 2001, Wu et al. found that incidence rates of proximal colon cancer among white men and women living in low-poverty counties were significantly higher compared to those living in high-poverty counties. No differences in subsite-specific rates were found for black men and women by county poverty level. In a study of CRC cases diagnosed in California from 1998 to 2002, Steinbrecher et al. found a strong association between census-tract SES and subsite-specific CRC incidence rates. Compared to the lowest SES neighborhoods, non-Hispanic whites (NHW) and non-Hispanic blacks (NWB) living in the highest SES neighborhoods had significantly lower rates of distal colon cancer, while only NHWs living in the highest SES neighborhoods had significantly lower rates of both proximal colon and rectal cancers. For Hispanics, the association between neighborhood SES and incidence of cancers of the proximal and distal colon and rectum was in the opposite direction compared to NHWs and NHBs.

Due to the inconsistency in study findings and a dearth of evidence, the SES contribution to subsite-specific incidence remains unclear. To further understand SES-based disparities in CRC incidence rates, we expand on the research by Wu et al. and Steinbrecher et al. by examining the associations between census-tract SES and CRC incidence and stage at diagnosis by anatomic subsite and race/ethnicity, using a population-based dataset covering about 42% of the US population. This study is the largest to date to examine these associations in the USA using a census-tract level measure of SES. 

## 2. Material and Methods

### 2.1. Study Population

The study population included 262,356 primary invasive and 15,741 primary* in situ* colon and rectal cases diagnosed from 2005 to 2009 from 15 state cancer registries (Arizona, Colorado, Connecticut, Florida, Georgia, Hawaii, Idaho, Iowa, Louisiana, Minnesota, New York, New Jersey, Texas, Utah, and West Virginia), and Los Angeles County. Each of these registries met the “Gold” or “Silver” certification levels by the North American Association of Central Cancer Registries (NAACCR) for data completeness and timeliness for these years. Each registry authorized the use of its data for this project, and the project was reviewed and approved by NAACCR's Institutional Review Board.

CRCs were classified into three anatomic subsites: proximal colon, distal colon, and rectum. Proximal colon cases included the following International Classification of Diseases for Oncology codes, third edition (ICD-O-3): cecum (C18.0); ascending colon (C18.2); hepatic flexure (C18.3); transverse colon (C18.4); and splenic flexure of colon (C18.5). Distal colon cases comprised descending colon (C18.6) and sigmoid colon (C18.7). Rectal cases included rectosigmoid junction (C19.9) and rectum not otherwise specified (C20.9). Cancers of the appendix (C18.1), colon not otherwise specified (C18.9), overlapping subsite (C18.8), and intestinal tract not otherwise specified (C26.0) accounted for 6% of all CRCs cases in the study and were included only in the analysis for all the CRCs combined, not in the analyses by subsite.

CRC screening in the average-risk population is recommended beginning at age 50 [15], so analysis of incidence rates by stage at diagnosis was limited to men and women aged 50 and older. CRC staging information was based on Surveillance Epidemiology and End Results (SEER) Summary Stage 2000 derived from collaborative stage variables. Cases diagnosed as* in situ* or at the localized stage were categorized as early-stage, and those diagnosed at either the regional or distant stage were categorized as late-stage. Cases with an unknown/missing stage, which accounted for 8.6% of the CRC cases among individuals 50 years and older, were excluded from analysis by stage. ICD-O-3 histology types leukemia and lymphoma (9590–9989), mesothelioma (9050–9055), and Kaposi sarcoma (9140) were also excluded.

### 2.2. Poverty, Ethnicity, and Population Data

All cases were geocoded by the individual registries to the 2000 census tracts and a census-tract poverty value was assigned to each case. Census tracts are defined by a partnership between the US Census Bureau and local authorities prior to each decennial census and are intended to include a relatively homogeneous population group of approximately 4,000 people. The census-tract poverty level was based on the poverty rate, the percentage of the population in a census-tract classified as being below the official poverty threshold according to the 2005–2009 American Community Survey (ACS) [16]. The poverty thresholds take into account family size and age composition (the number of children under 18) and inflation. For example, the official poverty threshold for a family of three with one child under 18 years of age was $17,268 in 2009. The Census Bureau does not adjust the poverty thresholds for regional or local variation in the cost of living. For this study census-tract poverty was grouped into four categories: <5%, 5%–<10%, 10%–<20%, and ≥20%. Approximately 3% of the cases did not have census tracts and/or poverty levels assigned and were excluded from the analysis. We chose poverty as the measure of area-based socioeconomic status because of the extensive literature using this measure [17] and because it was the sole SES variable available as part of the NAACCR Cancer in North America (CINA) dataset. Details about these data have been previously published [18, 19].

We used custom single-year sex and age-specific census-tract level residential population estimates for 2005–2009 developed by Woods & Poole Economics, Inc., for use by the SEER program. The Woods & Poole estimates were based on the assumption that the proportion of the population by age and sex for each tract in a particular county changed linearly from the 2000 to 2010 census. The total population for each county by age and sex was from the National Cancer Institute SEER population estimates database [20]. To estimate the proportion of the population in each tract for a particular county using the 2000 and 2010 censuses Woods & Poole used the 2000–2010 census-tract relationship file which indicates which tracts in the 2010 census relate to the tracts in the 2000 census. The Woods & Poole populations did not include information on race/ethnicity; therefore, to obtain these estimates we applied the census-tract race/ethnicity proportions from the 2010 census (Summary File 1, Tables PCT12H-PCT12O) to the Woods & Poole estimates, using a 2010-2000 census-tract crosswalk obtained from GeoLytics, Inc.

The data were grouped into Hispanic ethnicity plus four non-Hispanic racial groups: non-Hispanic white (NHW), non-Hispanic black (NHB), non-Hispanic American Indians and Alaska Natives (AI/AN), and non-Hispanic Asian or Pacific Islander (API). Non-Hispanics of multiple races were assigned to one of the four non-Hispanic groups based on the proportion of these groups in each census-tract; non-Hispanics of some other race were assigned to either AI/AN or API, based on the proportion of these two groups in each census-tract. This latter step was taken because these individuals tend to have origins in Central and South Asia and Central and South America. Multiple races and other race represented only 2.0% and 0.2%, of cases, respectively. Four race/ethnicity categories for both men and women were included in the final analysis: (1) NHW, (2) NHB, (3) Hispanic, and (4) API. AI/AN and cases with unknown race were excluded from the study due to small numbers, accounting for 0.25% and less than 1% of the total cases, respectively.

### 2.3. Statistical Analysis

Age-adjusted incidence rates (all ages included) and stage-specific age-adjusted incidence rates (ages ≥ 50 only) per 100,000 were computed by subsite (all CRC, proximal, distal, rectum), sex (men, women), race/ethnicity (NHW, NHB, Hispanic, API), and census-tract poverty categories. Only invasive cases were used to calculate the overall incidence rates to conform to standard population-based cancer incidence statistics in the USA, while incidence rates by stage included both invasive and* in situ* cases. The 2000 US standard population was used for age-adjustment of the rates. Incidence rate ratios (IRRs) were calculated for each of the three higher poverty categories versus the lowest poverty category. Confidence intervals (CIs) for incidence rates and IRRs were computed at the 95% level using the Tiwari et al. method, and the level of significance (alpha) was set at 0.05 (5%) for all statistical tests. Difference in the late-to-early stage rate ratios by poverty categories were tested with the 2-tailed *z*-statistic. For all analyses, we considered *P* values <0.05 to be statistically significant. Rate calculations were completed using SEER∗Stat software [21].

## 3. Results

The study population included 254,706 invasive CRC cases (all ages) and 223,015 invasive and* in situ* cases among persons aged 50 and older, diagnosed from 2005 to 2009. Proximal colon cases accounted for 42% of all CRCs; the distal colon, 24%; and the rectum, 28%. Among those aged 50+, the percentages of CRC by stage at diagnosis were 6%* in situ*, 38% localized, 31% regional, 17% distant, and 8% unknown. About 23% of the CRC cases were in the lowest poverty category (<5%), and 10% were in the highest poverty category (≥20%). Seventy-four percent of the study population was NHW; 12% NHB; 11% Hispanic; and 4% API.

### 3.1. CRC and Subsite-Specific Incidence by Poverty and Race/Ethnicity

Tables 1 and 2 show the CRC and subsite-specific age-adjusted IRRs by census-tract poverty and race/ethnicity for men and women, respectively. Incidence rates of CRC overall were significantly higher for men (IRR = 1.14 95% CI 1.12–1.17) and women (IRR = 1.06 95% CI 1.05–1.08) living in the highest (≥20%) poverty areas as compared to those living in the lowest poverty areas (<5%). Results for CRC overall were similar among NHW, NHB, and API men and women. Relative rates of CRC between the highest and lowest poverty category were greatest for NHB (IRR = 1.19 95% CI 1.12–1.28) and NHW (IRR = 1.18 95% CI 1.15–1.20) men (Tables 1 and 2). For Hispanics, an inverse association was observed with significantly lower IRs of CRC overall in the highest versus lowest poverty areas for both men (IRR = 0.88 95% CI 0.83–0.94) and women (IRR = 0.84 95% CI 0.79–0.89) (Tables 1 and 2).

By CRC subsite, differences in rates by poverty and race were most pronounced for male distal colon when compared to areas with the lowest poverty (5–9.9% poverty IRR = 1.04 95% CI 1.01–1.08; 10–19.9% poverty IRR = 1.12 95% CI 1.08–1.15; ≥20% poverty IRR = 1.24 95% CI 1.20–1.28). This pattern was largely driven by NHW, although NHB and API men in the highest poverty areas also had significantly higher incidence of distal colon cancer (NHB IRR = 1.29 95% CI 1.24–1.35; API IRR = 1.19 95% CI 1.02–1.40). Although similar patterns were found for distal colon cancer among all women, significant differences by poverty status were limited to NHW and NHB. Incidence rates were also significantly higher in the highest versus lowest poverty areas for rectal cancer among NHW and API men and women and NHB men. For Hispanics, an inverse association was observed with significantly lower IRs of proximal colon cancer in the highest versus lowest poverty areas. A similar pattern was found for rectal cancer among Hispanic women, but not Hispanic men (Tables 1 and 2).

### 3.2. CRC Incidence by Stage and Late versus Early-Stage IRRs

Patterns of CRC incidence by poverty level in stage-stratified analysis were similar to the overall rates (Table 3). Generally, NHW, NHB and API men and women living in the most impoverished areas had the highest late-stage CRC incidence rates. There was a significant inverse relationship between late-stage CRC incidence and poverty among Hispanic women as the IRR of late-stage CRC between those in the highest poverty areas versus the lowest poverty area was 0.80 (95% CI 0.73–0.87). This association was not seen among Hispanic men.

The CRC IRRs comparing late-to-early stage incidence by poverty level generally increased monotonically with increasing poverty for each of the race/ethnicity groups. However, only Hispanic and NHW men, and API women had significantly different late-to-early stage IRRs in the highest versus lowest poverty category. Among these groups, the greatest differences in IRRs between high and low poverty were found for API women (21% higher) and Hispanic men (11.8% higher).

**Figure 1:**
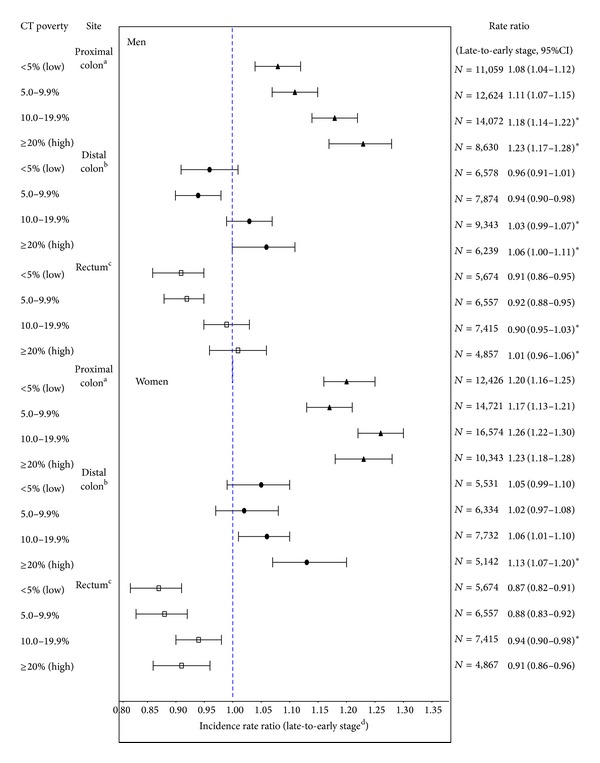
CRC subsite-specific incidence rate ratios and 95% CIs for late-to-early stage for selected areas in the United States (data are from selected population-based cancer registries that participate in the National Program of Cancer Registries (NPCR) and/or the Surveillance Epidemiology and End Results (SEER) Program: Arizona, Colorado, Connecticut, Florida, Georgia, Hawaii, Idaho, Iowa, Louisiana, Minnesota, New York, New Jersey, Texas, Utah, West Virginia, and Los Angeles). ^a^Proximal included ICD-O-3 codes C18.0, C18.2, C18.3, C18.4, and C18.5. ^b^Distal included ICD-O-3 codes 18.6 and C18.7. ^c^Rectum included ICD-O-3 codes C19.9 and C20.9. ^d^Late stage includes cases diagnosed at regional or distant stage; early stage includes cases diagnosed at* in situ* or localized stage.  CT, census tract; IRR, incidence rate ratio; CI, confidence interval; *N*, number of cases; rates are per 100,000 and age-adjusted to the 2000 US standard population.  *Statistically significant difference (*P* < 0.05) in late-to-early stage IRR by poverty level based on *z*-statistic. Difference is based on comparison of late-to-early stage IRRs for highest poverty categories with the lowest poverty category.

Figure 1 shows the subsite-specific late-to-early stage IRRs by poverty category. The late-to-early stage IRRs were highest for proximal colon and lowest for distal colon and rectum. The late-to-early stage IRRs for CRC subsites (proximal, distal, rectum) generally increased monotonically with increasing poverty, but the absolute differences in the late-to-early stage IRRs between the highest and lowest poverty category were greater for men than for women. For men, the late-to-early stage IRRs for each level of poverty were significantly higher in the highest two poverty categories compared to the lowest poverty category for each of the three subsites. For women, there was a significant increase in the late-to-early stage IRRs with increasing poverty for distal colon and rectum, but not proximal colon. Additional analyses stratified by race/ethnicity were not conducted because of small numbers.

## 4. Discussion

In this large population-based study, incidence rates of CRC were highest for NHW, NHB, and API men and women living in the most impoverished areas relative to the least impoverished areas. This finding is likely a result of higher rates of known risk factors of CRC among those living in the higher poverty areas, including an unhealthy diet, lack of physical activity, obesity, tobacco use [22, 23], and lower CRC screening rates [24–26]. Because CRC screening can identify cancers at the earliest stage and some screening modalities can also prevent cancer by detecting and removing precancerous polyps, screening can result in lower incidence overall among a highly screened group [15]. Endoscopy screening, which can decrease overall incidence as well as late-stage incidence, is less utilized by low income populations [27, 28]. Indeed, our stage-specific results were consistent with these patterns, as we report higher incidence rates of late-stage invasive CRC among populations living in the most impoverished areas. The higher incidence rates of CRC and late-stage disease among men and women living in the most versus least impoverished areas support the need to target socioeconomically disadvantaged areas for screening and develop programs to help reduce known modifiable risk factors for CRC like smoking and obesity.

Findings were generally consistent by anatomic subsite among NHW, NHB, and API men and women; however, the poverty gradients in incidence were stronger for distal colon and rectum than for proximal colon. These findings suggest that SES plays a more prominent role in distal colon and rectal cancers for these race groups than proximal colon cancers. This finding is also consistent with Doubeni et al. who examined data from the National Institutes of Health-AARP and also reported stronger SES gradients for cancers of the distal colon and rectum than for the proximal colon [29, 30]. These differences may reflect usage of endoscopy screening by poverty level and recent evidence indicating that colorectal cancer screening is more effective in reducing incidence in the rectum and distal colon than in the proximal colon [2, 31]. Because low income men and women have lower endoscopy screening rates compared to high income men and women [26], the greater effectiveness of screening for the distal colon and rectum may explain why the poverty gradients in incidence were stronger for these subsites compared to the proximal colon. The degree to which differences in screening prevalence and modality contributes to SES disparities in CRC incidence by subsite needs further study.

Among Hispanics an inverse relationship between poverty and CRC incidence rates was found, which is largely driven by the incidence of proximal colon cancer. This suggests that the risk profile for proximal colon cancer for Hispanics is different from the other groups in this study. The direction of the association for Hispanics is consistent with two other studies completed in the USA [12, 32] and resembles findings from European studies, which generally found that low-SES was associated with lower risk of colon and rectum cancers [11]. The source for this pattern remains unclear; however, acculturation may play a role. Acculturation is a process of adopting attitudes, values, and beliefs of a culture separate from the culture in which one was raised or educated [33]. Some risk factors for CRC among Hispanic women could vary according to their level of acculturation as studies suggest low SES Hispanics tend to be less acculturated and, therefore, might have certain behaviors that are protective against CRC [34]. A review study that assessed the relationship between acculturation and diet among Hispanics found that less acculturated Hispanics were generally of lower SES and consumed more fruit, rice, and beans but less sugar and sugar-sweetened beverages than more acculturated Hispanics consumed [35]. Studies have provided evidence that diets high in fruits or vegetables or fiber are potentially protective against CRC [36, 37]. Studies have also indicated that less acculturated Hispanics have lower smoking rates [38] and lower obesity prevalence compared to more acculturated Hispanics [39, 40], both risk factors for CRC [41, 42]. Despite the lower CRC incidence rates among Hispanics living in the highest poverty areas, the results from the stage analysis suggest that Hispanics living in high poverty are at increased risk of late-stage diagnosis, which seems to suggest a difference in access to health services and a need to enhance prevention activities in poor Hispanic neighborhoods. While our study did not examine the extent to which acculturation might contribute to the differences in incidence rates, previous studies do suggest that acculturation should be considered in developing health education messages and screening interventions that are culturally appropriate to the identified subpopulations [43, 44].

The late-stage incidence findings for non-Hispanic subgroups are consistent with previous studies [14, 45–47]. For all race/ethnicity groups, except Hispanic women, we found that the late-to-early stage IRRs for CRC were generally highest in the two highest poverty categories (≥20 and 10%–<20%) compared to the lowest poverty category (<5%). This is likely the result of both low screening rates and higher overall risk of CRC among those in the high poverty areas [46, 48]. This finding supports the need to continue to target socioeconomically disadvantaged areas for screening.

Our findings indicating higher late-to-early stage IRRs for proximal colon compared to distal colon regardless of poverty level were consistent with results from a previous US study that used county-based poverty measures [14]. Identifying higher late-to-early stage IRRs for proximal colon compared to the other subsites regardless of poverty presents an area of critical importance to cancer prevention and control because proximal colon cancer is not only increasing but it also generally has a worse prognosis regardless of stage [49]. Although colonoscopy provides a complete visual of the entire colon, it is most sensitive for identifying large, adenomatous polyps [50]. Serrated polyps, on the other hand, are more likely to be located in the proximal colon, and because they are flat, serrated polyps are more easily missed during colonoscopy [51]. From this perspective, our finding is provocative and supports clinical evidence that proximal colon cancers may be less preventable through colonoscopy screening than distal cancers [31, 52].

The finding indicating higher CRC incidence rates among APIs in the highest poverty category (≥20%) was not consistent with results from a previous study of California CRC patients, which found no association [12]. This inconsistency may be due to the different study time periods, the use of a different SES measure or regional differences in the API study populations in regard to ethnicity, nativity, primary language spoken, SES, and level of acculturation, which may influence attitudes toward preventive screening and affect CRC risk [12]. The API subgroups included in both studies were extremely different. Compared to California, a greater proportion of API cases included in this study were Asian Indian or Pakistani (8.3% versus 3.5%), Hawaiian (4.7% versus 0.78%), and Japanese (19.2% versus 14.2%) [53]. Because cancer incidence, screening practices, and risk factors for API subgroups (e.g., Hawaiian, Chinese, Korean) vary considerably, future work should consider examining subsite-specific CRC incidence by SES for the different API subgroups.

Our study has several limitations that should be considered. First, we did not have individual-level SES data, information about screening, nor risk factor data, which limited our ability to consider important health behaviors and confounders that may explain the relationships observed in this study. Second, we excluded cases with unknown stage from the stage-stratified analysis of CRC incidence. Stage at diagnosis is often missing in population-based cancer data and was missing for 8.6% of the cases in our study [54, 55]. This may introduce bias into our study because it is systematically missing—particularly for older patients, blacks, and the more impoverished. Unknown stage also has a poor prognosis (33.2% 5-year survival rate compared with a 90% for a local stage, 71% for a regional stage, and 12.9% for a distant stage) [56]. This potential selection bias limits the external validity (generalizability) of our findings as our sample likely has a higher proportion of cases diagnosed at regional or distant stage. Furthermore, because cases with unknown stage are more likely to come from impoverished areas, by excluding these cases in our stage analysis by poverty, we likely underestimated the CRC incidence rates for the most impoverished. There is currently no standard method for handling unknown stage cases in cancer surveillance, and studies often simply exclude these cases as we did [45, 46, 57]. Future work should include methods to include these data, such as through imputation [58], to improve the precision and reliability of the association between area-based SES and CRC stage. A third limitation is related to the assignment of race/ethnicity of the cases which is based primarily on information from the medical record. Race/ethnicity from the medical record may be based on self-identification by the patient, assigned by a health care provider or an admissions clerk, or assigned indirectly based on birthplace, surname, or maiden name. It is unknown how often race/ethnicity is assigned by either self-report or another approach; however misclassification could impact the accuracy of the incidence rates since the denominator data is based on self-report to the US Census. Fourth, the intercensal populations between 2000 and 2010 were estimated using linear interpolation and race/ethnicity specific populations were based on the proportion of these groups in 2010. If our estimates vary significantly from the actual populations, this will also impact the accuracy of incidence rates. Finally, our decision to proportionally allocate the small percentage of non-Hispanics of multiple races (2.0%) and of some other races (0.2%) could also impact the accuracy of the incident of rates if the population estimates are different than the actual populations. Allocating or bridging multiracial respondents in the Census to single-race categories remains a critical research area and attention must be paid to the allocation decisions as it could result in either overestimating or underestimating rates. A study by Mays et al. demonstrated that the effect of different allocation methods on the estimation of race/ethnicity associated health disparities varies across the different types of health outcomes assessed [59]. Strengths of our study include the large multiethnic population, representing 15 states and the most populous, ethnically diverse county in the USA (Los Angeles County), and the use of census-tract level poverty as our unit of measure of area-based SES, which is a more homogenous measure of area-based poverty than county.

## 5. Conclusion

Overall, this study showed that there was a significantly higher incidence of CRC and subsite-specific rates among those living in the most impoverished areas compared to those living in the least impoverished areas for all race/ethnicity groups, except for Hispanics, for whom an inverse relationship was observed. The disparity in incidence of CRC by poverty was more pronounced for rectal and distal colon cancer than for proximal colon cancers. Furthermore, we also observed that higher poverty increased the risk of late-stage and, consequently, less survivable disease. Because those living in the poorest areas have the highest rates of CRC incidence and a higher proportion of late-stage diagnoses, there are important public health implications for this study for the burden of CRC in the USA. It is likely that screening is an important moderator to the relationship of area-based SES and CRC stage at diagnosis. Because CRC screening remains our most effective prevention tool for both CRC incidence and mortality developing culturally appropriate screening programs in impoverished neighborhoods to ensure residents have access to screening may help ameliorate race and ethnic disparities in CRC incidence and mortality. However, this must also be dovetailed with public health programs to reduce obesity and other risk factors for CRC in these neighborhoods. Effective programs that improve diet and lower obesity may reduce the risk of CRC overall, including lowering rates of proximal CRC. Efforts that promote healthy behavior and ensure access to CRC screening according to national guidelines will help reduce socioeconomic disparities in CRC incidence and stage at diagnosis.

## Figures and Tables

**Table 1:** Overall CRC and subsite-specific age-adjusted incidence rates^a^ for men by poverty and race/ethnicity for selected areas^b^ in the United States, 2005–2010.

CT poverty	CRC^c^			Proximal colon^d^			Distal colon^e^			Rectum^f^		
	*N*	IR (95% CI)	IRR	*N*	IR (95% CI)	IRR	*N*	IR (95% CI)	IRR	*N*	IR (95% CI)	IRR
All men												
<5% (low)	29,704	49.6 (49–50.2)	Ref	11,563	20.0 (19.6–20.4)	Ref	7,117	11.7 (11.4–12.0)	Ref	9,185	14.8 (14.4–15.1)	Ref
5.0–9.9%	34,495	49.9 (49.4–50.5)	1.01 (0.99–1.02)	13,338	19.8 (19.5–20.1)	0.99 (0.97–1.01)	8,523	12.2 (11.9–12.5)	**1.04 (1.01–1.08) **	10,576	14.9 (14.6–15.2)	1.01 (0.98–1.04)
10.0–19.9%	39,863	52.5 (51.9–50.0)	**1.06 (1.04–1.07)**	14,933	20.1 (19.8–20.5)	1.01 (0.98–1.03)	10,052	13.1 (12.8–13.3)	**1.12 (1.08–1.15) **	12,225	15.7 (15.4–16.0)	**1.07 (1.05–1.09) **
20+ (high)	26,224	56.6 (55.9–57.3)	**1.14 (1.12–1.17)**	9,301	20.8 (20.4–21.2)	**1.04 (1.01–1.07) **	6,813	14.5 (14.2–14.9)	**1.24 (1.20–1.28) **	8,156	17.0 (16.6–17.4)	**1.15 (1.12–1.19) **
NHW men												
<5% (low)	25,419	49.9 (49.3–50.5)	Ref	10,083	20.3 (19.9–20.7)	Ref	6,015	11.7 (11.4–12.0)	Ref	7,752	14.8 (14.5–15.1)	Ref
5.0–9.9%	28,514	50.5 (49.9–51.1)	1.01 (1.00–1.03)	11,241	20.2 (19.8–20.5)	0.99 (0.96–1.02)	6,954	12.2 (11.9–12.5)	**1.05 (1.01–1.09)**	8,615	15.1 (14.8–15.4)	1.02 (0.99–1.05)
10.0–19.9%	29,532	53.5 (52.9–54.1)	**1.07 (1.05–1.09)**	11,318	20.6 (20.2–21.0)	1.01 (0.99–1.04)	7,298	13.1 (12.8–13.4)	**1.13 (1.09–1.17)**	8,973	16.2 (15.8–16.5)	**1.09 (1.06–1.13)**
20+ (high)	12,764	58.8 (57.8–59.8)	**1.18 (1.15–1.20)**	4,632	21.5 (20.9–22.2)	**1.06 (1.02–1.10)**	3,288	15.1 (14.5–15.6)	**1.29 (1.24–1.35)**	3,937	18.0 (17.4–18.6)	**1.22 (1.17–1.27)**
NHB men												
<5% (low)	1,262	57.3 (53.8–60.9)	Ref	515	25.0 (22.6–27.5)	Ref	312	14.1 (12.4–15.9)	Ref	343	13.7 (12.1–15.4)	Ref
5.0–9.9%	2,143	54.2 (51.7–56.8)	0.95 (0.88–1.02)	883	23.1 (21.4–24.8)	0.92 (0.82–1.05)	511	12.8 (11.6–14.1)	0.91 (0.78–1.06)	610	14.4 (13.2–15.7)	1.05 (0.91–1.22)
10.0–19.9%	4,199	57.9 (56.0–59.8)	1.01 (0.94–1.08)	1,693	24.5 (23.3–25.8)	0.98 (0.88–1.10)	1,007	13.7 (12.8–14.7)	0.97 (0.85–1.12)	1,166	14.6 (13.7–15.5)	1.07 (0.93–1.22)
20+ (high)	6,955	68.3 (66.6–70.0)	**1.19 (1.12–1.28)**	2,752	27.9 (26.8–29.0)	**1.12 (1.01–1.24)**	1,730	16.8 (15.9–17.6)	**1.19 (1.04–1.36)**	1,913	17.9 (17.0–18.7)	**1.31 (1.15–1.49)**
Hispanic men												
<5% (low)	1,564	51.0 (48.2–53.8)	Ref	549	19.4 (17.7–21.2)	Ref	366	11.7 (10.4–13.1)	Ref	529	15.7 (14.3–17.2)	Ref
5.0–9.9%	2,526	45.8 (43.9–47.8)	**0.90 (0.84–0.96)**	864	16.6 (15.4–17.8)	**0.86 (0.76–0.84)**	675	12.0 (11.1–13.1)	1.03 (0.89–1.18)	830	14.2 (13.2–15.2)	0.90 (0.80–1.02)
10.0–19.9%	4,597	46.4 (45.0–47.9)	**0.91 (0.85–0.97)**	1,510	16.4 (15.5–17.3)	**0.84 (0.76–0.94)**	1,269	12.4 (11.7–13.2)	1.06 (0.93–1.21)	1,506	14.3 (13.5–15.1)	0.91 (0.82–1.02)
20+ (high)	5,540	44.8 (44.8–46.1)	**0.88 (0.83–0.94)**	1,673	14.4 (13.6–15.1)	**0.74 (0.67–0.83)**	1,505	12.1 (11.4–12.7)	1.03 (0.91–1.17)	1,933	14.8 (14.1–15.6)	0.94 (0.85–1.05)
API men												
<5% (low)	1,459	39.5 (37.4–41.8)	Ref	416	12.5 (11.3–13.8)	Ref	424	11.4 (10.3–12.6)	Ref	561	14.1 (12.9–15.3)	Ref
5.0–9.9%	1,312	40.2 (38.0–42.6)	1.02 (0.94–1.10)	350	11.5 (10.3–12.8)	0.92 (0.79–1.07)	383	11.8 (10.6–13.1)	1.04 (0.89–1.20)	521	15.1 (13.8–16.5)	1.07 (0.95–1.22)
10.0–19.9%	1,535	43.7 (41.4–46.0)	**1.10 (1.02–1.19)**	412	12.3 (11.1–13.6)	0.98 (0.85–1.13)	478	13.6 (12.4–14.9)	**1.19 (1.04–1.37)**	580	15.9 (14.5–17.3)	1.13 (1.00–1.28)
20+ (high)	965	45.1 (42.3–48.1)	**1.14 (1.05–1.24)**	244	11.9 (10.4–13.5)	0.95 (0.80–1.12)	290	13.6 (12.1–15.3)	**1.19 (1.02–1.40)**	373	16.9 (15.2–18.7)	**1.20 (1.05–1.38)**

^a^Rates are per 100,000 persons and age-adjusted to the 2000 US standard population.

^b^Data are from selected population-based cancer registries that participate in the National Program of Cancer Registries (NPCR) and/or the Surveillance Epidemiology and End Results (SEER) Program: Arizona, Colorado, Connecticut, Florida, Georgia, Hawaii, Idaho, Iowa, Louisiana, Minnesota, New York, New Jersey, Texas, Utah, West Virginia, and Los Angeles.

^c^Colon and rectum (CRC) included ICD-O-3 codes C18.0–C18.9, C19.9, C20.9, and C26.0.

^d^Proximal included ICD-O-3 codes C18.0, C18.2, C18.3, C18.4, and C18.5.

^e^Distal included ICD-O-3 codes 18.6 and C18.7.

^f^Rectum included ICD-O-3 codes C19.9 and C20.9.

IR: incidence rate; IRR: incidence rate ratio; CI: confidence interval; CT: census tract; NHW: non-Hispanic white; NHB: non-Hispanic black; API: Asian Pacific Islander.

Bold numbers indicate significant associations *P* < 0.05; reference (<5% below poverty).

**Table 2:** Overall CRC and subsite-specific age-adjusted incidence rates^a^ for women by poverty and race/ethnicity for selected areas^b^ in the United States, 2005–2010.

CT poverty	CRC^c^			Proximal colon^d^			Distal colon^e^			Rectum^f^		
	*N*	IR (95% CI)	IRR	*N*	IR (95% CI)	IRR	*N*	IR (95% CI)	IRR	*N*	IR (95% CI)	IRR
All women												
<5% (low)	28,398	38.3 (37.8–38.8)	Ref	13,086	17.6 (17.3–17.9)	Ref	6,225	8.4 (8.2–8.7)	Ref	6,969	9.4 (9.2–9.7)	Ref
5.0–9.9%	33,062	38.2 (37.8–38.6)	1.00 (0.98–1.01)	15,523	17.7 (17.4–18.0)	1.00 (0.98–1.03)	7,113	8.3 (8.1–8.5)	0.98 (0.95–1.02)	8,101	9.5 (9.3–9.7)	1.01 (0.98–1.04)
10.0–19.9%	38,041	39.2 (38.8–39.6)	**1.02 (1.01–1.04)**	17,586	17.8 (17.6–18.1)	1.01 (0.99–1.04)	8,622	9.0 (8.8–9.2)	**1.07 (1.03–1.10)**	9,088	9.6 (9.4–9.8)	1.01 (0.98–1.05)
20+ (high)	24,919	40.7 (40.2–41.2)	**1.06 (1.05–1.08)**	10,994	17.8 (17.5–18.2)	1.01 (0.99–1.04)	5,823	9.6 (9.4–9.9)	**1.14 (1.10–1.18)**	6,127	10.1 (9.9–10.4)	**1.07 (1.03–1.10)**
NHW women												
<5% (low)	24,316	38.8 (38.3–39.3)	Ref	11,432	18.0 (17.6–18.3)	Ref	5,156	8.3 (8.1–8.6)	Ref	5,891	9.6 (9.3–9.8)	Ref
5.0–9.9%	27,041	38.6 (38.1–39.1)	1.00 (0.98–1.01)	12,981	17.9 (17.6–18.3)	1.00 (0.97–1.03)	5,649	8.3 (8.1–8.5)	0.99 (0.96–1.03)	6,517	9.7 (9.5–9.9)	1.01 (0.97–1.05)
10.0–19.9%	28,063	40.0 (39.5–40.5)	**1.03 (1.01–1.05)**	13,441	18.4 (18.1–18.7)	1.02 (1.00–1.05)	6,101	9.0 (8.8–9.2)	**1.08 (1.04–1.12)**	6,530	9.9 (9.6–10.1)	1.03 (0.99–1.07)
20+ (high)	12,074	42.5 (41.7–43.3)	**1.10 (1.07–1.12)**	5,582	18.6 (18.1–19.1)	1.04 (1.00–1.07)	2,652	9.8 (9.5–10.2)	**1.18 (1.12–1.24)**	2,896	10.9 (10.5–11.3)	**1.14 (1.09–1.19)**
NHB women												
<5% (low)	1,292	43.6 (41.2–46.1)	Ref	605	21.1 (19.4–22.9)	Ref	292	9.5 (8.4–10.7)	Ref	308	10.0 (8.9–11.2)	
5.0–9.9%	2,385	43.9 (42.1–45.8)	1.01 (0.94–1.08)	1,118	21.2 (20.0–22.5)	1.01 (0.91–1.11)	524	9.3 (8.5–10.2)	0.98 (0.85–1.14)	562	9.9 (9.1–10.8)	0.99 (0.86–1.15)
10.0–19.9%	4,625	45.3 (44.0–46.7)	1.04 (0.98–1.11)	2,099	21.0 (20.1–22.0)	1.00 (0.91–1.09)	1,099	10.6 (9.9–11.2)	1.11 (0.97–1.28)	1,051	10.0 (9.4–10.6)	1.00 (0.88–1.14)
20+ (high)	7,342	48.7 (47.5–49.8)	**1.12 (1.05–1.19)**	3,342	22.3 (21.6–23.1)	1.06 (0.97–1.16)	1,703	11.2 (10.7–11.7)	**1.18 (1.04–1.34)**	1,707	11.1 (10.6–11.7)	1.11 (0.98–1.27)
Hispanic women												
<5% (low)	1,500	36.8 (34.9–38.7)	Ref	607	16.2 (14.9–17.6)	Ref	359	8.2 (7.3–9.1)	Ref	411	9.2 (8.3–10.2)	Ref
5.0–9.9%	2,361	33.9 (32.5–35.3)	**0.92 (0.86–0.99)**	991	15.0 (14.1–16.0)	0.93 (0.83–1.03)	567	7.8 (7.2–8.5)	0.96 (0.83–1.10)	612	8.3 (7.6–9.0)	0.90 (0.79–1.02)
10.0–19.9%	3,979	31.9 (30.9–32.9)	**0.87 (0.82–0.92)**	1,577	13.3 (12.6–14.0)	**0.82 (0.74–0.90)**	1,003	7.7 (7.2–8.2)	0.94 (0.83–1.07)	1,088	8.4 (7.9–8.9)	0.91 (0.81–1.03)
20+ (high)	4,611	30.8 (29.9–31.7)	**0.84 (0.79–0.89)**	1,773	12.5 (11.9–13.1)	**0.77 (0.70–0.85)**	1,209	7.7 (7.3–8.2)	0.95 (0.84–1.07)	1,245	8.0 (7.5–8.5)	**0.87 (0.77–0.98)**
API women												
<5% (low)	1,290	28.6 (27.0–30.2)	Ref	442	10.4 (9.4–11.4)	Ref	418	8.9 (8.0–9.8)	Ref	359	7.7 (6.9–8.6)	Ref
5.0–9.9%	1,275	30.1 (28.4 –31.8)	1.05 (0.97–1.14)	433	10.6 (9.6–11.7)	1.02 (0.89–1.17)	373	8.5 (7.7–9.4)	0.96 (0.83–1.11)	410	9.5 (8.6–10.5)	**1.23 (1.06–1.43)**
10.0–19.9%	1,374	30.6 (28.9–32.2)	1.07 (0.99–1.16)	469	11.0 (10.0–12.1)	1.06 (0.92–1.21)	419	9.1 (8.3–10.1)	1.03 (0.89–1.18)	419	8.9 (8.0–9.8)	1.15 (0.99–1.33)
20+ (high)	892	32.1 (30.0–34.3)	**1.12 (1.03–1.23)**	297	10.8 (9.6–12.1)	1.04 (0.89–1.21)	259	9.3 (8.2–10.5)	1.04 (0.89–1.22)	279	10.0 (8.8–11.2)	**1.29 (1.10–1.52)**

^a^Rates are per 100,000 persons and age-adjusted to the 2000 US standard population.

^b^Data are from selected population-based cancer registries that participate in the National Program of Cancer Registries (NPCR) and/or the Surveillance Epidemiology and End Results (SEER) Program: Arizona, Colorado, Connecticut, Florida, Georgia, Hawaii, Idaho, Iowa, Louisiana, Minnesota, New York, New Jersey, Texas, Utah, West Virginia, and Los Angeles.

^c^Colon and rectum (CRC) included ICD-O-3 codes C18.0–C18.9, C19.9, C20.9, and C26.0.

^d^Proximal included ICD-O-3 codes C18.0, C18.2, C18.3, C18.4, and C18.5.

^e^Distal included ICD-O-3 codes 18.6 and C18.7.

^f^Rectum included ICD-O-3 codes C19.9 and C20.9.

IRR: incidence rate ratio; CI: confidence interval; CT: census tract; NHW: non-Hispanic white; NHB: non-Hispanic black; API: Asian Pacific Islander.

Bold numbers indicate significant associations *P* < 0.05; reference (<5% below poverty).

**Table 3:** CRC incidence rates for early and late stage tumors and late-to-early stage incidence rate ratios for men and women 50 years+ by race/ethnicity and poverty for selected areas in the United States^a^.

CT poverty	CRC^b^ early stage^c^			CRC^b^ late stage^d^			late-to-early stage IRR
	*N*	Rate (95% CI)	IRR	*N*	Rate (95% CI)	IRR	
All men							
<5% (low)	13,102	79.2 (77.8–80.6)	Ref	13,419	81.1 (79.7–82.5)	Ref	1.02 (1.00–1.05)
5.0–9.9%	14,969	78.2 (76.9–79.4)	0.99	15,556	80.8 (79.5–82.1)	1.00	1.03 (1.01–1.06)
10.0–19.9%	16,546	79.1 (77.9–80.3)	1.00	18,497	87.6 (86.4–88.9)	**1.08** ∗	**1.11 (1.08**–**1.13)** ^†^
20+ (high)	10,492	83.6 (82–85.2)	**1.06** ∗	12,097	95.2 (93.5–96.9)	**1.17** ∗	**1.14 (1.11**–**1.17)** ^†^
NHW men							
<5% (low)	11,350	79.5 (78.0–81.0)	Ref	11,542	80.9 (79.4–82.4)	Ref	1.02 (0.99–1.04)
5.0–9.9%	12,637	79.3 (77.9–80.7)	1.00	12,907	80.9 (79.5–82.3)	1.00	1.02 (0.99–1.04)
10.0–19.9%	12,604	80.7 (79.3–82.2)	1.02	13,759	87.7 (86.2–89.2)	**1.08** ∗	**1.09 (1.06**–**1.11)** ^†^
20+ (high)	5,388	88.4 (86.0–90.8)	**1.11** ∗	5,924	96.5 (94.0–99.0)	**1.19** ∗	**1.09 (1.05**–**1.13)** ^†^
NHB men							
<5% (low)	505	86.5 (78.4–95.3)	Ref	576	100.7 (91.8–110.2)	Ref	1.16 (1.02–1.33)
5.0–9.9%	836	80.9 (75–87.2)	0.94	957	92.1 (85.9–98.8)	0.92	1.14 (1.03–1.26)
10.0–19.9%	1,628	85.4 (80.9–90)	0.99	1,929	99.2 (94.4–104.1)	0.99	1.16 (1.08–1.25)
20+ (high)	2,700	97.8 (94–101.8)	**1.13** ∗	3,265	117.8 (113.5–122.1)	**1.17** ∗	1.20 (1.14–1.27)
Hispanic men							
<5% (low)	619	79.4 (72.8–86.4)	Ref	657	82.6 (75.9–89.7)	Ref	1.04 (0.92–1.17)
5.0–9.9%	978	69.4 (64.8–74.2)	**0.87** ∗	1,102	77.2 (72.5–82.2)	0.93	1.11 (1.01–1.22)
10.0–19.9%	1,716	67.2 (63.8–70.6)	**0.85** ∗	2,098	80.9 (77.2–84.6)	0.98	**1.20 (1.12**–**1.29)** ^†^
20+ (high)	2,034	64.4 (61.5–67.4)	**0.81** ∗	2,470	76.3 (73.2–79.5)	0.92	**1.18 (1.11**–**1.26)** ^†^
API men							
<5% (low)	628	63.4 (58.2–68.9)	Ref	644	65.7 (60.4–71.3)	Ref	1.04 (0.92–1.17)
5.0–9.9%	518	59.7 (54.5–65.4)	0.94	590	67.0 (61.5–73.0)	1.02	1.12 (0.99–1.27)
10.0–19.9%	598	63.4 (58.3–68.9)	1.00	711	75.2 (69.5–81.1)	**1.14** ∗	1.18 (1.06–1.33)
20+ (high)	370	64.1 (57.7–71.1)	1.01	438	75.7 (68.6–83.2)	**1.15** ∗	1.18 (1.02–1.36)
All women							
<5% (low)	11,712	56.9 (55.8–57.9)	Ref	13,140	63.4 (62.3–64.5)	Ref	1.12 (1.09–1.14)
5.0–9.9%	13,681	56.4 (55.5–57.4)	0.99	15,226	62.2 (61.2–63.2)	0.98	1.10 (1.08–1.13)
10.0–19.9%	15,301	56.2 (55.3–57.1)	0.99	17,915	65.4 (64.4–66.4)	**1.03** ∗	**1.16 (1.14**–**1.19)** ^†^
20+ (high)	9,879	58.2 (57.0–59.4)	1.02	11,577	67.9 (66.6–69.1)	**1.07** ∗	**1.17 (1.14**–**1.20)** ^†^
NHW women							
<5% (low)	10,113	57.3 (56.1–58.4)	Ref	11,294	63.6 (62.4–64.8)	Ref	1.11** **(1.08–1.14)
5.0–9.9%	11,339	56.6 (55.6–57.7)	0.99	12,572	62.3 (61.2–63.5)	0.98	1.10 (1.07–1.13)
10.0–19.9%	11,544	57.4 (56.3–58.4)	1.00	13,401	66.3 (65.2–67.5)	**1.04** ∗	**1.16 (1.13**–**1.19)** ^†^
20+ (high)	4,994	60.8 (59.1–62.6)	**1.06** ∗	5,642	69.1 (67.3–71)	**1.09** ∗	1.14 (1.09–1.18)
NHB women							
<5% (low)	534	66.6 (60.9–72.6)	Ref	580	73.1 (67.1–79.4)	Ref	1.10 (0.97–1.24)
5.0–9.9%	941	64.3 (60.2–68.7)	0.97	1,041	72.1 (67.7–76.7)	0.99	1.12 (1.02–1.23)
10.0–19.9%	1,748	63.3 (60.3–66.4)	0.95	2,145	77.4 (74.1–80.8)	1.06	1.22 (1.15–1.30)
20+ (high)	2,914	69.5 (67–72.1)	**1.04** ∗	3,419	81.8 (79–84.6)	**1.12** ∗	1.18 (1.12–1.24)
Hispanic women							
<5% (low)	534	50.6 (46.3–55.2)	Ref	684	65.8 (60.8–71)	Ref	1.30 (1.16–1.46)
5.0–9.9%	896	48.9 (45.7–52.3)	0.97	1,036	56.6 (53.1–60.2)	**0.86** ∗	1.16 (1.06–1.27)
10.0–19.9%	1,477	44.8 (42.6–47.2)	**0.89** ∗	1,760	53.4 (50.9–56)	**0.81** ∗	1.19 (1.11–1.28)
20+ (high)	1,654	41.9 (39.9–44)	**0.83** ∗	2,078	52.3 (50.1–54.7)	**0.80** ∗	1.25 (1.17–1.33)
API women							
<5% (low)	531	44.2 (40.5–48.3)	Ref	582	48.2 (44.2–52.3)	Ref	1.09 (0.96–1.23)
5.0–9.9%	505	43.6 (39.8–47.7)	0.99	577	50.5 (46.4–54.9)	1.05	1.16 (1.02–1.31)
10.0–19.9%	532	43.5 (39.8–47.4)	0.98	609	49.3 (45.4–53.5)	1.02	1.13 (1.01–1.28)
20+ (high)	317	41.2 (36.8–46)	0.93	438	56.7 (51.5–62.3)	**1.18** ∗	**1.38 (1.19**–**1.60)** ^†^

^a^Data are from selected population-based cancer registries that participate in the National Program of Cancer Registries (NPCR) and/or the Surveillance Epidemiology and End Results (SEER) Program: Arizona, Colorado, Connecticut, Florida, Georgia, Hawaii, Idaho, Iowa, Louisiana, Minnesota, New York, New Jersey, Texas, Utah, West Virginia, and Los Angeles.

^b^Colon and rectum (CRC) included ICD-O-3 codes C18.0–C18.9, C19.9, C20.9, and C26.0.

^c^Early (*in situ* and localized).

^d^Late (regional and distant).

CRC: colon and rectum cancer; IRR: incidence rate ratio; CI: confidence interval; NHW: non-Hispanic white; NHB: non-Hispanic black; API: Asian Pacific Islander.

∗Statistically significant IRR (*P* < 0.05); reference (<5% below poverty).

^†^Statistically significant difference (*P* < 0.05) in late-to-early stage IRR by poverty level is based on *z*-statistic. Difference is based on comparison of late-to-early stage IRRs for the two highest poverty categories with the lowest poverty category (<5% below poverty).
